# Markers of Epstein–Barr Virus Infection in Association with the Onset and Poor Control of Rheumatoid Arthritis: A Prospective Cohort Study

**DOI:** 10.3390/microorganisms11081958

**Published:** 2023-07-31

**Authors:** Danijela Miljanovic, Andja Cirkovic, Ivica Jermic, Milica Basaric, Ivana Lazarevic, Milka Grk, Rada Miskovic, Aleksa Despotovic, Ana Banko

**Affiliations:** 1 Institute of Microbiology and Immunology, Faculty of Medicine, University of Belgrade, 11000 Belgrade, Serbia; ivana.lazarevic@med.bg.ac.rs (I.L.); ana.banko@med.bg.ac.rs (A.B.); 2 Institute for Medical Statistics and Informatics, Faculty of Medicine, University of Belgrade, 11000 Belgrade, Serbia; andja.cirkovic@med.bg.ac.rs (A.C.); alexadespotovic21@gmail.com (A.D.); 3 Institute of Rheumatology, Faculty of Medicine, University of Belgrade, 11000 Belgrade, Serbia; ivicaje@yahoo.com (I.J.); basaric.milica@gmail.com (M.B.); 4 Institute of Human Genetics, Faculty of Medicine, University of Belgrade, 11000 Belgrade, Serbia; milkagrk@gmail.com; 5 Clinic of Allergy and Immunology, University Clinical Center of Serbia, 11000 Belgrade, Serbia; rada_delic@hotmail.com; 6 Faculty of Medicine, University of Belgrade, 11000 Belgrade, Serbia

**Keywords:** Epstein–Barr virus (EBV), rheumatoid arthritis (RA), biomarker, anti-EBV antibodies, EBNA1 variants

## Abstract

Although the connection between Epstein–Barr virus (EBV) and rheumatoid arthritis (RA) has been studied for over 40 years, many questions still need clarification. The study aimed to analyze the possible association between anti-EBV antibody titers, EBV DNA viremia, EBV infection status and EBNA1 (Epstein–Barr nuclear antigen 1—EBNA1) variants and clinical parameters of RA patients. This prospective cohort study included 133 RA patients and 50 healthy controls. Active/recent EBV infection was more prevalent in RA patients than in controls (42% vs. 16%, *p* < 0.001). RA patients had higher titers of anti-EBV-CA-IgM (capsid antigen—CA) and anti-EBV-EA(D)-IgG (early antigen—EA) antibodies than controls (*p* = 0.003 and *p* = 0.023, respectively). Lower levels of anti-EBNA1-IgG and anti-EBV-CA-IgG were observed in RA patients who received methotrexate (anti-EBNA1 IgG *p* < 0.001; anti-EBV-CA IgG *p* < 0.001). Based on amino acid residue on position 487, two EBNA1 prototypes were detected: P-Thr and P-Ala. Patients with active/recent EBV infection had a five times more chance of having RA and a nearly six times more chance of getting RA. Also, EBV active/recent infection is twice more likely in newly diagnosed than in methotrexate-treated patients. Further studies are needed to clarify “who is the chicken and who is the egg” in this EBV–RA relationship.

## 1. Introduction

Rheumatoid arthritis is an autoimmune disease characterized by chronic inflammation and destruction of joints. The worldwide prevalence of RA is around 1%, two to four times more common in women than men leading to reduced life expectancy and overall quality of life [1]. Synovial hyperplasia, joint destruction and the production of autoantibodies such as rheumatoid factors (RFs) and anti-citrullinated protein antibodies (ACPAs) are hallmarks of the disease [2]. The etiology of RA still needs to be wholly resolved. It is a multifactorial disease involving complex interactions between genetic and environmental factors [3]. Motifs QKRAA, QRRAA and RRRAA at positions 70–74 of the third hypervariable region within human leukocyte antigen DRB1 (HLA-DRB1) alleles are called “shared epitopes” and represent the most important genetic factors for the susceptibility and severity to RA [4]. Epstein–Barr virus (EBV) is a ubiquitous environmental factor linked to RA. Over 90% of the human population is infected with EBV by the age of forty [5], and EBV establishes lifelong latency in memory B lymphocytes after primary infection. EBV can exhibit three latency states (I, II, III) with expression of a limited number of genes, including Epstein–Barr nuclear antigen (*EBNA1*) [5]. EBV can switch from latent to lytic phase and reactivate in both immunocompetent and immunocompromised individuals. Different viral proteins are produced during reactivation, including early antigen (EA) [6], a DNA polymerase accessory protein that initiates viral replication and the lytic phase of EBV infection in B cells [2]. Different serological profiles could be related to latent and lytic stages of EBV infection. Anti-EBV-CA (capsid antigen—CA) and anti-EA antibodies of IgM and IgG classes are produced during the primary infection. In contrast, anti-EBNA1 antibodies are detected during recovery and the late primary infection disease [7]. Anti-EBNA1 and anti-EBV-CA IgG antibodies imply past EBV infection, while anti-EA antibodies indicate viral replication [7].

The role of EBV in the etiopathogenesis of RA is still an open question without an adequate answer. It has been hypothesized that EBV may cause RA through several mechanisms [8]. The most known and well described is the mechanism of molecular mimicry. More than forty years ago, Alspaugh and Tan reported that sera from RA patients were reactive against RA nuclear antigen in EBV-transformed lymphocytes [9,10]. EBV protein gp110 has sequence homology with the shared epitopes (QKRAA motif) of HLA-DR4, and EBV-infected people have antibodies and T cells that recognize the QKRAA motif within both gp110 and HLA-DR4 [5,11]. Also, antibodies against the p107, the major epitope of EBNA1 protein, bind to collagen and keratin [12]. Additionally, RA patients have impaired control of EBV infection due to chronic stimulation of B cells [13]. Several studies have reported elevated antibody levels against EBV antigens such as VCA, EA and EBNA1 in RA patients compared to controls [9,10,14]. On the other hand, a meta-analysis by Ball et al., done in 2015, failed to show a significant association between RA and anti-EBV-CA, anti-EBV-EBNA1 and anti-EBV-EA IgG [8]. In addition, RA patients have a ten-fold higher EBV DNA load in peripheral mononuclear cells (PMNCs), synovium and saliva, as well higher presence of viral proteins and higher numbers of circulating EBV-infected cells compared to controls subject [15,16,17].

Data regarding the genetic diversity of EBNA1 variants among RA patients are missing. To our knowledge, only one study by Masuoka et al. in 2018 describes variants of EBNA1 protein in RA [18]. There is still no clear evidence about the possible role of mutations and variants of EBNA1 in the pathogenesis of RA.

Although the connection between EBV and RA has been studied for over 40 years, many questions still need clarification. No data about EBV prevalence in the Serbian cohort of RA patients are available. Recently, the so-called preclinical period of RA has been studied a lot by scientists. In addition to the use of ACPAs and RF as preclinical markers of RA, identifying and stratifying EBV-associated risk factors that are present and detectable before the clinical onset of RA and of poor RA control may help us better understand the mechanisms of RA development and progression. These EBV biomarkers might modify the course of RA with appropriate therapy. We aimed to elucidate the role of EBV infection in newly diagnosed RA patients and methotrexate-treated RA patients who did not achieve an adequate response. Also, we wanted to analyze the possible association between anti-EBV antibody titers, EBV DNA viremia and EBNA1 variants and the most significant clinical parameters of RA patients. Our study’s novelty represents data about the genetic variability of EBNA1 in RA patients.

## 2. Materials and Methods

### 2.1. Study Design and Participants

This prospective cohort study included 133 RA patients treated at the Institute of Rheumatology in Belgrade between June 2020 and November 2021. All of them fulfilled the American College of Rheumatology (ACR)/EULAR criteria for diagnosis of RA [19]. Detailed history, physical examination, joint ultrasound, X-ray imaging of hands and feet and relevant laboratory analysis according to local protocols were performed for each participant. The control group included 50 participants without systemic autoimmune disease and had a negative family history of inflammatory rheumatic diseases. Before any procedures, all participants signed written informed consent. The study was performed under the Declaration of Helsinki and was approved by the Ethical Board of the Faculty of Medicine, University of Belgrade and the Ethical Board of the Institute of Rheumatology (No 1550/IX-14 and 19/1-31).

### 2.2. Samples

RA patients’ blood samples were collected at the Institute of Rheumatology in Belgrade. Control group blood samples were obtained from volunteers at the Institute of Rheumatology, Belgrade and the Institute of Microbiology and Immunology, Faculty of Medicine, University of Belgrade. Five to ten milliliters of blood were collected from each participant. One test tube was with a clot activator, and the serum was separated after centrifugation. The second test tube was with ethylenediaminetetraacetic acid (EDTA), and after centrifugation for 15 min at 3000× *g*, the plasma was separated. Sera and plasma from each patient were placed into sterile tubes and stored at −70 °C until further analysis.

### 2.3. Determination of Anti-EBV Antibodies Presence and Titer

Anti-EBV-CA IgM and IgG, anti-EBV-EA IgG and IgM and anti-EBNA1 IgG were identified and measured using commercial ELISAs according to the manufacturer’s instructions in collected sera (Euroimmun, Lubeck, Germany). Standard calibrators were used in each assay to calculate index values/optical density (OD) ratios, which serve as a quantitative measure of IgG antibody titers or a semi-quantitative measurement of IgM antibody levels. All assays met pre-determined quality control measures based on positive, negative and blank controls. The positivity of IgG antibody presence was defined by a cut-off value of 20 relative units (RU/mL). The positivity of IgM antibody presence was defined as OD ratio ≥ 1.1. Absorbances were recorded on a Multiskan FC microplate reader (Thermo Scientific, Waltham, MA, USA) using a wavelength of 405 nm with background subtraction at 650 nm. The lower detection limit of anti-EBNA1 IgG was 0.9 RU/mL with a linearity range from 7–176 RU/mL. The anti-EBV-EA(D) IgG linearity range was 2–158 RU/mL with the lower detection limit of 0.8 RU/mL. The lower detection limit of anti-EBV-CA IgG was 0.9 RU/mL with a linearity range from 4–141 RU/mL. The lower detection limit of the anti-EBV-CA IgM and anti-EBV-EA(D) IgM is a ratio of 0.08.

### 2.4. Viral DNA Extraction, Amplification and Quantification of EBV DNA

According to the manufacturer’s instructions, viral DNA was isolated from 200 μL plasma using a PureLink Genomic DNA Mini Kit (Invitrogen by Thermo Fisher Scientific, Waltham, MA, USA). Nested PCR methods were performed to amplify two EBV genes: *EBNA1* and *LMP1*. Primers for detecting and amplifying C-terminal regions of *EBNA1* and *LMP1* genes are presented in Table 1.

Amplification of both regions of EBV was carried out in thermocycler Master Cycler Gradient (Eppendorf, Germany) in a total reaction volume of 25 µL. The first round of the nested PCR thermal cycling program for amplification of the *EBNA1* gene consisted of the following steps: initial denaturation at 95 °C for 3 min, followed by 40 cycles at 95 °C for 30 s, 57 °C for 1 min, 72 °C for 1 min, and final extension at 72 °C for 10 min; and in the second round of nested PCR annealing temperature was 60 °C. The thermal cycling program for amplifying the *LMP1* gene was the same in both rounds of nested PCR. It consisted of the following steps: an initial denaturation at 95 °C for 3 min, followed by 40 cycles at 95 °C for 30 s, 58 °C for 1 min, 72 °C for 1 min, and final extension at 72 °C for 10 min.

Visualization of PCR products of appropriate length (329 bp for *EBNA1* and 506 bp for *LMP1*) was performed by electrophoresis in 2% agarose gel stained with SYBR Safe DNA gel stain (Invitrogen, Waltham, MA, USA).

Quantification of all positive EBV DNA samples was carried out. Primers, probes and protocols used for EBV quantification were previously described in Jakovljevic et al., 2018 [22]. Results were expressed as copies of viral DNA per milliliters of blood.

### 2.5. Sequencing of EBV EBNA1 and LMP1 Genes

All positive PCR products were purified using QIAGEN MinElute Purification Kit (QIAGEN, Hilden, Germany), according to the manufacturer’s instructions. Isolates were directly sequenced using Big Dye Terminator v3.1 Cycle Sequencing Kit (Applied Biosystems, Foster City, CA, USA) and inner primers in an automatic sequencer (ABI PRISM 310 Genetic Analyzer; Applied Biosystems, Foster City, CA, USA).

The sequence of each EBNA1 isolate was compared to EBV reference sequences in Bioedit 7.0.1 software [23] to identify EBNA1 prototypes and subtypes. Determination of EBNA1 variants was done manually by searching for signature amino acid changes at the following positions: 471, 475, 476, 479, 487, 492, 499, 500, 502, 517, 520, 524, 525, 528, and 533.

The EBNA1 sequence obtained in this study is deposited in the GenBank database under the following accession numbers: OR271568-OR271572.

### 2.6. Data Analysis

Numerical data were described with the arithmetic mean and standard deviation or median and range, depending on the data distribution. Normal distribution was evaluated by mathematical (Kolmogorov–Smirnov and Shapiro–Wilk test, skewness and kurtosis, and coefficient of variation) and graphical methods (histogram, box-plot, Q-Q diagram). Categorical variables were presented as absolute and relative numbers in the form of n (%). Student *t*-test for two independent samples or Mann–Whitney U test was used to compare numerical data between study cohorts, depending on the data distribution. The Chi-square test was used to test the difference in the distribution of categories of two independent samples.

To evaluate all possible factors influencing a person’s probability of having rheumatoid arthritis, univariate logistic regression analysis was performed first, with multivariate analysis after that with significant variables from the previous analysis in the model. VIF method and correlation coefficients were used to evaluate multicollinearity. All variables with VIF > 5 were eliminated from the multivariate model. Also, according to the covariance matrix and correlation coefficients, one of two highly correlated variables with a higher *p*-value in univariate logistic regression was eliminated. The forward Wald regression method was applied, and only the last step was presented in the results. The odds ratio (OR), 95% confidence interval of odds ratio (95% CI OR), and *p*-value were reported.

## 3. Results

### 3.1. Characteristics of the Study Participants

A total of 133 RA patients and 50 healthy controls were included in this study. RA patients were subdivided into two groups: RA A and RA B group. RA A included 80 newly diagnosed RA patients, and the RA B group included 53 methotrexate-treated RA patients who did not respond adequately to therapy. RA patients were significantly older than controls (58.86 ± 11.78 vs. 45.40 ± 8.78, *p* < 0.001) with predominantly the female gender in both groups (88% vs. 72%, *p* = 0.024) and higher frequency of tertiary or higher educational level in controls than in RA patients (75% vs. 23%, *p* < 0.001). There were significantly more smokers with longer smoking duration in RA A than in the RA B group (*p* = 0.002 and *p* = 0.012, respectively). Detailed socio-demographic, clinical and laboratory characteristics of study participants are shown in Table 2.

### 3.2. Status and Markers of EBV Infection in RA Patients and Controls

EBV DNA was detected in 9/133 (7%) RA patients and in none of the controls 0/50 (0%). Seven out of nine positive EBV DNA patients were positive on both genes (*EBNA1* and *LMP1*), while two patients were positive only in the *LMP1* gene. In the RA A group, 7/80 (9%) patients were EBV DNA positive, while in RA B, only 2/53 (4%) patients, and there was no statistically significant difference between RA A and RA B subgroups (*p* = 0.263) nor in comparison with controls (*p* = 0.059). EBV viral load was determined in all nine positive EBV DNA samples. The number of copies of EBV DNA per milliliters of blood ranged from 100 to 1800, with an average load of 428.8 copies/mL. Anti-EBV-CA IgM and anti-EBV-EA(D) IgG antibodies were more prevalent in RA patients than in controls (19% vs. 2%, *p* = 0.003 and 19% vs. 6%, *p* = 0.025, respectively). There was no difference in the prevalence of any of the evaluated anti-EBV antibodies between the RA A and RA B subgroups. The status of EBV infection was defined based on the results from EBV DNA and anti-EBV antibody testing. The active/recent EBV infection was defined if EBV DNA and/or anti-EBV IgM and/or anti-EA(D) IgG antibodies were present. Active/recent EBV infection was more prevalent in RA patients than in controls (42% vs. 16%, *p* < 0.001) and in RA A than in the RA B subgroup (*p* = 0.009) (Table 3).

**Figure 1:**
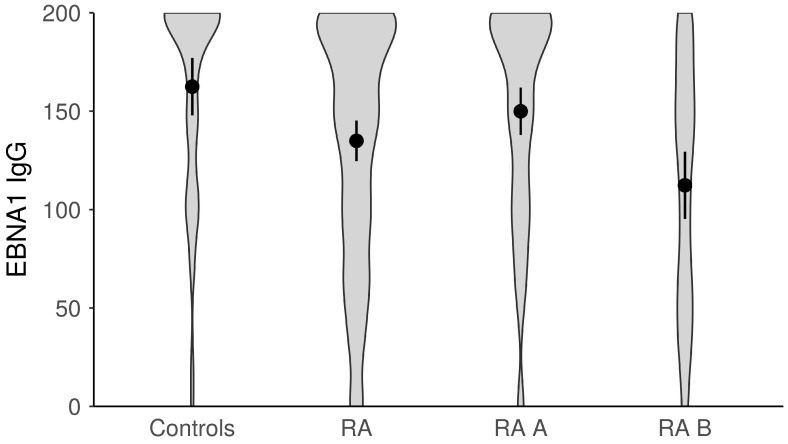
The titers of anti-EBV-EBNA1 IgG antibodies in study groups (dots represent means and whiskers 95% of the mean). EBNA1—Epstein–Barr nuclear antigen; RA—all rheumatoid arthritis patients; RA A subgroup—newly diagnosed RA patients; RA B subgroup—methotrexate-treated RA patients.

**Figure 2:**
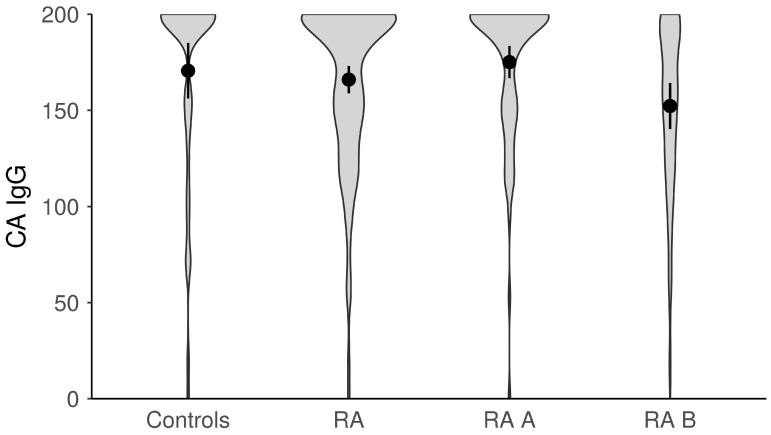
The titers of anti-EBV-CA IgG antibodies in study groups (dots represent means and whiskers 95% of the mean). CA—capsid antigen; RA—all rheumatoid arthritis patients; RA A subgroup—newly diagnosed RA patients; RA B subgroup—methotrexate-treated RA patients.

**Figure 3:**
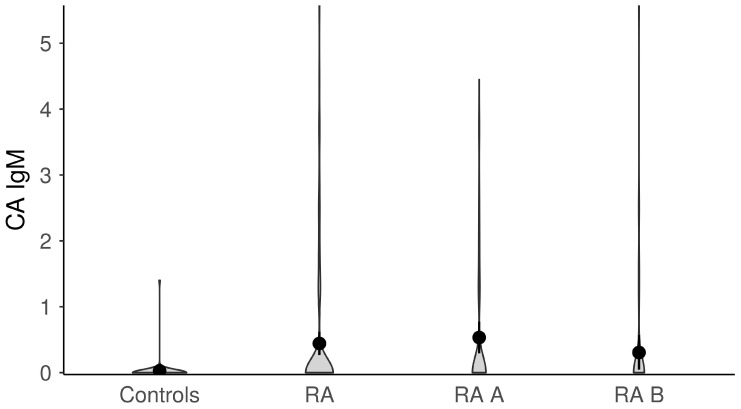
The titers of anti-EBV-CA IgM antibodies in study groups (dots represent means and whiskers 95% of the mean). CA—capsid antigen; RA—all rheumatoid arthritis patients; RA A subgroup—newly diagnosed RA patients; RA B subgroup—methotrexate-treated RA patients.

**Figure 4:**
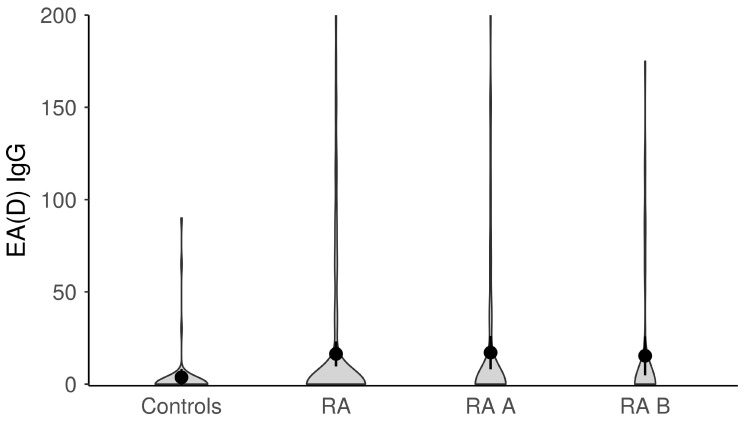
The titers of anti-EBV-EA(D) IgG antibodies in study groups (dots represent means and whiskers 95% of the mean). EA(D)—early antigen (diffuse); RA—all rheumatoid arthritis patients; RA A subgroup—newly diagnosed RA patients; RA B subgroup—methotrexate-treated RA patients.

**Figure 5:**
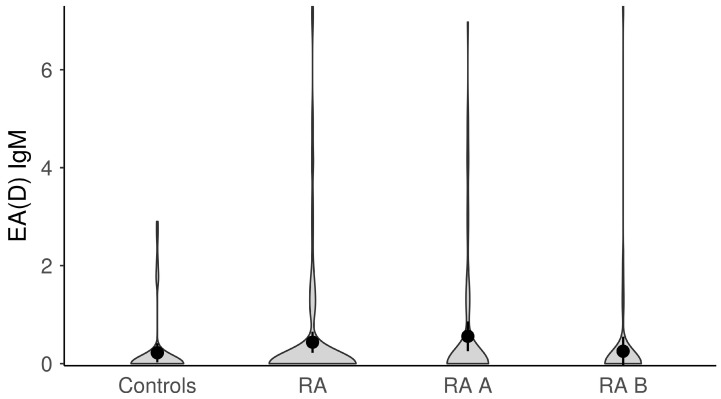
The titers of anti-EBV-EA(D) IgM antibodies in study groups (dots represent means and whiskers 95% of the mean). EA(D)—early antigen (diffuse); RA—all rheumatoid arthritis patients; RA A subgroup—newly diagnosed RA patients; RA B subgroup—methotrexate-treated RA patients.

The titers of anti-EBV antibodies in the evaluated study groups and subgroups were analyzed afterward (Figure 1, Figure 2, Figure 3, Figure 4 and Figure 5). RA patients had significantly higher titers of anti-EBV-CA IgM and anti-EBV-EA(D) IgG antibodies than controls (*p* = 0.003 and *p* = 0.023, respectively), whilst titers of anti-EBV-EBNA1-IgG were significantly higher in controls than in RA patients (*p* = 0.001). RA patients and controls did not differ according to the titers of anti-EBV-CA IgG and anti-EBV-EA(D) IgM antibodies (*p* = 0.104 and *p* = 0.335, respectively). It was also determined that the titer of anti-EBV-EBNA1 IgG antibodies was significantly higher in RA A than in RA B patients (*p* < 0.001).

### 3.3. Association of Anti-EBV Antibodies Titers and RA Activity/Severity, Inflammation and Immunological Parameters in RA Patients

We analyzed whether there is an association between the titers of anti-EBV antibodies and clinical parameters of disease activity/severity, inflammation and immunological parameters (DAS28, CDAI, SDAI, RAID, HAQ, CRP, ANA, ACPA, RF and anticardiolipin IgG antibodies). A negative mild statistically significant association between anti-EBNA1 antibodies titer and CDAI (ρ = −0.171; *p* = 0.049) was found. The lower the titer of anti-EBNA1 antibodies, the higher the CDAI score was. A positive moderate statistically significant association was found in newly discovered RA patients (RA A group) between titers of anti-EBNA1 IgG and RF (ρ = 0.317; *p* = 0.004). The higher the titers of anti-EBNA1 antibodies, the higher the levels of RF were. There was no association between other anti-EBV antibodies and other investigated parameters in the RA cohort.

### 3.4. Association of Anti-EBV Antibodies Titers and Therapy in RA Patients

Since RA therapy includes immunosuppressive drugs that can influence the production of anti-EBV antibodies, we analyzed the possible association between anti-EBV antibodies and RA therapy used in this study. There was no statistically significant difference in the titers of anti-EBV antibodies and the use of NSAID and corticosteroids (results not shown). Lower levels of anti-EBNA1 IgG and anti-EBV-CA IgG were observed in RA patients who received MTX (anti-EBNA1 IgG *p* < 0.001; anti-EBV-CA IgG *p* < 0.001). Also, lower levels of anti-EBV-CA IgG were established in RA patients treated with sulfasalazine (*p* = 0.016). On the other hand, RA patients treated with paracetamol had higher levels of anti-EBV-CA IgM than those who did not receive this medication (*p* = 0.046).

### 3.5. EBV Viremic vs. Non-Viremic RA Patients and Disease Characteristics

EBV viremic and non-viremic RA patients significantly differed according to the level of RF (*p* = 0.003) (Table 4). Higher titers of RF were detected in RA patients with positive EBV DNA in the blood.

### 3.6. EBNA1 Variants in RA Patients

Of 7 EBNA1 positive samples, the EBNA1 sequence was obtained from 5 samples. Two different variants of EBNA1 were detected, P-thr and P-ala, based on specific amino acid residue on position 487 of EBNA1. P-thr was detected in 80% of isolates, while one EBNA1 sequence belonged to P-ala. That one EBNA1 P-ala sequence was classified as a sub-variant named P-ala-sv-2 because it had two amino acid residues at positions 499 and 524, different from prototype subtype P-ala EBV B95.8 (Table 5).

### 3.7. The Impact of EBV Infection on Disease Onset, Having Disease and Poor Disease Control

Finally, we evaluated the possible association between markers of EBV infection and having RA, the onset of RA and poor RA control. The results of multivariate logistic regression modeling are presented in Table 6. We found that active/recent EBV infection was a factor independently associated with having rheumatoid arthritis (OR = 4.645, 95%CI OR = 1.33–16.19, *p* = 0.016), a risk factor for the onset of RA (RR = 5.47, 95%CI RR = 1.28–23.35, *p* = 0.022), and a characteristic twice more probable in newly diagnosed than in poorly controlled RA patients (OR = 0.451, 95%CI OR = 0.21–0.98, *p* = 0.045), regardless of the age, gender, educational level, smoking status and family history for RA.

## 4. Discussion

The role of EBV in RA is still unresolved, complex and involves several mechanisms. This study assessed possible associations between EBV infection status, presence and titers of anti-EBV antibodies, genetic EBNA1 variants and RA development and progression. We included newly diagnosed and methotrexate-treated RA patients and performed a comprehensive serological, molecular and genetic analysis of EBV with a clinical assessment of each patient. Previous studies have demonstrated that RA patients have more EBV-infected B cells and a 10-fold systemic EBV overload than healthy controls [15]. Also, using immunosuppressive drugs such as methotrexate is associated with a suspected increased risk of developing EBV-associated lymphomas in RA patients [24]. This study detected EBV DNA in 7% of RA patients and none of the healthy controls. Our study’s low prevalence of EBV DNA may result from using the conventional qPCR method for detection and using plasma instead of PBMCs. Kuusela et al., 2018 used a novel digital droplet PCR and found that the frequency of EBV DNA increased from 15.2% to 35.5% in RA patients during follow-up [25]. Digital droplet PCR is a method with a low detection limit and is highly sensitive, with the possibility to detect the presence of just a few DNA molecules [25]. Also, RA patients with EBV reactivation may have a low viremia, and ddPCR may increase the chance of detecting all the patients with cell-free EBV DNA due to EBV lytic infection. In our study in newly diagnosed RA, viremia was detectible in 9%, while in methotrexate-treated patients in 4%. The average viremia was around 400 copies per milliliter of blood. This result is in accordance with the findings of two prospective cohorts [24,26] that long-term use of methotrexate decreases EBV load over time in patients with RA.

Statistically higher titers and percentages of positive anti-EBV-CA IgM and anti-EBV-EA(D)-IgG antibodies were detected in RA patients compared with controls. On the other hand, titers of anti-EBNA1 IgG were significantly higher in controls than in RA patients. EBV EA is a marker of lytic EBV infection, and its higher titers in RA patients indicate some degree of EBV reactivation. Also, in our study of newly discovered RA patients, anti-EBV-EA(D)-IgG antibody titers were higher than in methotrexate-treated RA patients, although a statistically significant difference was not found. The increased titers of IgG antibodies against EBV EA in RA patients are in accordance with results from previous studies [6,27]. Also, a possible trigger for these elevated anti-EA titers can be immunosuppressive therapy. However, higher titers of anti-EA antibodies were also found in patients with systemic sclerosis and primary anti-phospholipid syndrome that do not use immunosuppressive therapy [28]. Increased levels of anti-EA are most likely the result of an interaction between viral lifecycle and altered immune state in RA patients unrelated to immunosuppressive therapy [6]. In one of the newest studies done by Fechtner et al., 2022 authors found that higher levels of anti-EBV-EA(D)-IgG antibodies in preclinical RA cases suggested an association between EBV reactivation and future development of RA [29]. All these different findings may indicate that lytic EBV infection influences RA or that RA influences the shift from latent to lytic EBV infection. The question of who is older, the “chicken or egg”, can also be applied here. Further prospective cohort studies are needed to comprehensively analyze the role of the presence and elevated titers of anti-EBV-EA(D) IgG antibodies in RA and the possibility of them becoming viral markers for RA development and progression.

EBNA1 is the most important protein for maintaining the EBV genome during the latent phase of the EBV cycle, and antibodies against EBNA1 are markers of past infection and persist for life [2]. Anti-EBNA1 antibodies are a strong risk factor in developing multiple sclerosis [30]. Results from some previous studies have reported elevated levels of IgG-EBNA1 antibodies in RA patients [2,29]. However, in our study, decreased titers of anti-EBNA1 were found in the RA cohort compared to controls. Also, newly discovered RA patients had higher titers of anti-EBNA1 than methotrexate-treated RA patients. Our results are in accordance with the results of some previously published studies [9,10,31]. Svendsen et al. [32] found that IgG-EBNA1 antibody level was higher in healthy co-twins from RA twin pairs but not in RA-affected twins, and the Swedish population-based Epidemiological Investigation of RA (EIRA) cohort had lower levels of IgG-EBNA1, especially in ACPA positive RA patients [33]. These different findings regarding levels of anti-EBNA1 antibodies indicate that the humoral immune response against EBV infection is compromised and dysregulated in patients with autoimmune diseases. The limitations of this study regarding anti-EBNA1 were that we only tested for IgG antibody levels, and no other EBNA1 antibody isotypes were included. On the other hand, Svendsen et al. [32] found that increased IgM EBNA1 is associated with RA and RA predisposition.

The anti-EBV-CA IgM titers were also elevated in our cohort of RA patients compared to healthy controls. These antibodies are also markers of lytic EBV infection, and increased levels of anti-EBV-CA IgM could indicate a possible host attempt to keep lytic EBV infection under control.

We also analyzed the possible association between anti-EBV antibody titers and RA activity, inflammation, immunological parameters and therapy in RA patients. In newly discovered RA patients, a positive association was observed between higher titers of anti-EBNA1 IgG and higher titers of RF. The hypothesis may be that the same process is responsible for initializing and producing EBNA1 antibodies and RF [2]. In RA patients with positive EBV DNA in blood, higher titers of RF were detected with statistically significant differences in comparison with non-viremic RA patients. It has been demonstrated that RF could activate B cells and trigger the lytic infection of EBV in them [34]. Regarding therapy, lower levels of anti-EBNA1 IgG and anti-EBV-CA IgG were observed in RA patients treated with MTX. Also, sulfasalazine was associated with lower levels of anti-EBV-CA IgG. Anti-EBNA1 IgG and anti-EBV-CA IgG without IgM are typical markers of past EBV infection. The titers remain relatively the same during the whole life. The known fact is that immunosuppressive states induced in different ways can decrease the titers of these antibodies during life. Since RA patients are immunosuppressed due to the therapy used, it is not surprising that we detected decreased levels of anti-EBNA1 and anti-EBV-CA IgG in this study. No other association between anti-EBV titers and investigated parameters was found.

The major novelty of this study is the analysis of EBNA1 sequences among Serbian RA patients. The aim was to determine if EBNA1 variants play a role in RA development. EBNA1 is the only latent protein expressed constantly, even in EBV-associated carcinoma [35]. There are two prototypes of EBNA1: P and V that are further classified based on the AA present at codon 487 into P-alanine (P-ala), P-threonine (P-thr), V-proline (V-pro), V-leucine (V-leu) and V-valine (V-val) [36]. Some studies indicate that specific EBNA1 variants polymorphisms play an important role in EBV-associated malignancies [37,38]. Only the P prototype was detected in our study among RA patients. The most dominant subvariant was P-thr, which was detected in 80% of EBNA1-positive RA patients. One patient with the P-ala sequence was classified as sub-variant P-ala-sv-2. This sub-variant has already been identified among Serbian but also Danish patients with nasopharyngeal carcinoma, lymphoma and healthy subjects [39,40] and is a European-related subvariant of EBNA1 [36]. In the only study done before ours that investigated the sequence variability of EBNA1 in RA patients, the predominant EBNA1 prototype is prototype V [18]. Since this study was done in Japan, the dominance of the V prototype is expected, and they did not find that EBNA1 gene variants are risk factors for RA. Due to the low number of obtained EBNA1 sequences, adequate statistical analysis could not be done to get a clearer picture of the possible role of the EBNA1 sequence in RA development.

## 5. Conclusions

There are some similarities between EBV infection and RA. They are both chronic, with episodes of activation. The criteria for determining the status of EBV infection was defined by using both serological and molecular findings in this study. The active/recent EBV infection, defined by the positive EBV DNA and/or presence of anti-EBV IgM and/or EA(D) IgM/EA(D)IgG, was more prevalent in RA patients than in controls (42.1% vs. 16%). More than half (51.25%) of newly diagnosed RA patients had active EBV infection, while one-third (28.3%) had it in methotrexate-treated RA patients. Multivariant logistic regression modeling showed that active/recent EBV infection was an independent factor associated with having rheumatoid arthritis, a risk factor for the onset of RA adjusted for age, gender, education level, smoking status and family history of RA. Patients with active/recent EBV infection had five times more chance of having RA and nearly six times more chance of getting RA. Also, EBV active/recent infection was probably twice more in those newly diagnosed than in poorly controlled methotrexate-treated patients.

Our study demonstrates elevated titers of anti-EBV-CA IgM and anti-EBV-EA(D)-IgG antibodies, more prevalent active EBV infection in RA patients than controls. Studies with larger plasma samples are needed to assess the clinical utility of these antibodies and determine the possible threshold of these elevated antibodies for estimating RA activity. Still, there is yet to be a consensus on which molecular technique to use and what is the most representative sample for EBV DNA detection in this group of patients. Although our findings indicate that active/recent EBV infection may contribute to having RA or the onset of RA, further studies are needed to clarify “who is the chicken and who is the egg” in this EBV RA relationship.

## Figures and Tables

**Table 1:** Primers used in this study for the amplification and sequencing of the EBV, *LMP1* and *EBNA1* genes.

Primers	Sequence (5′-3′)	Nucleotide Position ^a^	Reference
***LMP1* gene**			
Fw1 (outer)	GCTAAGGCATTCCCAGTAAA	168081–168100	Li et al., 2009 [20]
Rev1 (outer)	GATGAACACCACCACGATG	168726–168774	Li et al., 2009 [20]
Fw2 (inner)	CGGAACCAGAAGAACCCA	169679–169658	Li et al., 2009 [20]
Rev2 (inner)	TCCCGCACCCTCAACAAG	168702–168719	Li et al., 2009 [20]
***EBNA1* gene**			
Fw1 (outer)	AGATGGTGAGCCTGACGTG	109218–109236	Lorenzetti et al., 2010 [21]
Rev1 (outer)	GCATCCTTCAAAACCTCAGC	109663–109682	Lorenzetti et al., 2010 [21]
Fw2 (inner)	CCCGCAGATGACCCAGGAGA	109261–109280	Lorenzetti et al., 2010 [21]
Rev2 (inner)	GGGTCCAGGGGCCATTCCAAA	109570–109590	Lorenzetti et al., 2010 [21]

^a^ Location of primers is relative to EBV B95.8 reference sequencing. LMP1—latent membrane protein; EBNA1—Epstein–Barr nuclear antigen.

**Table 2:** Baseline characteristics of the study participants.

Characteristic	Group		*p* *	Subgroups		*p* *
	RA n = 133	Control n = 50		RA A n = 80	RA B n = 53	
***Sociodemographics***						
**Age (years), mean ± sd**	58.86 ± 11.78	45.40 ± 8.78	**<0.001 £**	59.45 ± 12.73	57.96 ± 10.21	0.478 £
**Gender, n (%)**						
Male	37 (27.8)	6 (12.0)	**0.024 §**	23 (28.7)	14 (26.4)	0.769 §
Female	96 (72.2)	44 (88.0)		57 (71.3)	39 (73.6)	
**Educational level, n (%)**						
Primary	25 (18.8)	1 (2.0)	**<0.001 §**	14 (17.5)	11 (20.8)	0.058 §
Secondary	78 (58.6)	11 (22.4)		53 (66.3)	25 (47.2)	
Tertiary or higher	30 (22.6)	37 (75.5)		13 (16.3)	17 (32.1)	
**BMI, mean ± sd**	25.21 ± 4.32	26.12 ± 4.49	0.213 £	25.34 ± 4.34	25.03 ± 4.34	0.684 £
**Smoking status, n (%)**						
Smoker	63 (47.4)	26 (52.0)	0.576 §	51 (63.7)	19 (35.8)	**0.002 §**
Non-smoker	70 (52.6)	24 (48.0)		29 (36.3)	34 (64.2)	
**Smoking duration (years), med (min–max)**	7.0 (0.0–60.0)	10.0 (0.0–35.0)	0.494 ¥	20.0 (0–60.0)	0.0 (0.0–53.0)	**0.012 ¥**
***Disease characteristics***						
**Duration of RA (years), med (min–max)**	8.0 (1.0–40.0)	/	NA	/	8.0 (1.0–40.0)	NA
**Family history of RA, n (%)**	54 (40.6)	1 (2.0)	**<0.001 §**	32 (40.0)	22 (41.5)	0.862 §
**VAS, med (min–max)**						
Patient	60 (10–100)	/	NA	60 (10–100)	70 (30–100)	0.168 ¥
Physician	50 (10–90)	/	NA	50 (10–90)	50 (10–90)	0.917 ¥
Pain	60 (10–90)	/	NA	60 (10–100)	60 (30–90)	0.694 ¥
**Ultrasound examination, med (min–max)**						
Total number of swollen joints	7 (0–32)	/	NA	6 (0–32)	9 (0–30)	**<0.001 ¥**
Total number of tender joints	11 (0–33)	/	NA	10 (0–33)	14 (0–31)	**<0.001 ¥**
**RA activity/severity**						
DAS28 (SE), mean ± sd	5.24 ± 1.12	/	NA	4.96 ± 1.33	5.67 ± 0.42	**<0.001 £**
DAS28 (CRP), mean ± sd	4.72 ± 1.10	/	NA	4.47 ± 1.23	5.10 ± 0.75	**<0.001 £**
CDAI, med (min–max)	18 (1–40)	/	NA	12 (1–26)	22 (7–40)	**<0.001 §**
SDAI, med (min–max)	16 (0–63)	/	NA	12.3 (0–63)	22.8 (6–62)	**<0.001 §**
RAID, med (min–max)	5 (1–18)	/	NA	5 (1–16)	5 (1–18)	0.351 §
RAQoL, med (min–max)	12 (1–28)	/	NA	10.25 (1–28)	13.0 (1–27)	**0.017 §**
HAQ, med (min–max)	1.125 (0.125–2.625)	/	NA	0.935 (0.125–2.125)	1.250 (0.280–2.625)	**<0.001 §**
ESR, med (min–max)	35 (6–120)	/	NA	37 (6–100)	34 (10–120)	0.711 §
CRP, med (min–max)	15 (0–152.4)	/	NA	12.8 (0–84.1)	19.3 (1.7–152.4)	**0.042 §**
**Immunological parameters**						
ANA, med (min–max)	640.0 (40.0–640.0)	/	NA	20.0 (0.0–640.0)	40.0 (0.0–640.0)	0.984 §
aCL-IgM, med (min–max)	0.0 (0.0–7.4)	/	NA	0.0 (0.0–1.0)	0.0 (0.0–31.2)	0.590 §
aCL-IgG, med (min–max)	0.0 (0.0–15.1)	/	NA	0.0 (0.0–7.8)	0.0 (0.0–15.1)	0.138 §
RF, med (min–max)	151 (0.0–641.6)	/	NA	127.5 (2.0–641.6)	162.0 (0.0–500.0)	0.198 §
ACPA, med (min–max)	320.0 (0.0–500.0)	/	NA	284.5 (3.5–500.0)	350.0 (0.0–500.0)	0.866 §
**Current RA therapy**						
Corticosteroids, n (%)	75 (56.4)	/	NA	35 (43.8)	40 (75.5)	**<0.001 §**
Antimalarials, n (%)	25 (18.8)	/	NA	0 (0.0)	25 (47.2)	**<0.001 §**
Sulfasalazine, n (%)	9 (6.8)	/	NA	0 (0.0)	9 (17.0)	**<0.001 §**
Methotrexate (MTX)	52 (39.1)	/	NA	0 (0.0)	52 (98.1)	**<0.001 §**
Paracetamol, n (%)	53 (39.8)	/	NA	30 (37.5)	23 (43.4)	0.497 §
NSAID	106 (57.9)	/	NA	59 (73.8)	47 (88.7)	**0.036 §**
**Comorbidities, n (%)**						
AH	61 (45.9)	10 (20.0)	**0.001 §**	36 (45.0)	25 (47.2)	0.806 §
DM	18 (13.5)	2 (4.0)	0.065 §	11 (13.8)	7 (13.2)	0.929 §
Cardiovascular events (CVI, TIA, MI, AP)	37 (27.8)	1 (2.0)	**<0.001 §**	24 (30.0)	13 (24.5)	0.491 §

* for the level of significance of 0.05, according to Student’s *t*-test £, Chi-square test §, Mann–Whitney U test ¥. NA—not applicable only for RA patients with inadequate disease control. Abbreviations: RA—rheumatoid arthritis; BMI—body mass index; VAS—visual analog score; min—minimum; max—maximum; med—median; DAS28—disease activity score; SE—sedimentation; CRP—C reactive protein; CDAI—clinical disease activity index; SDAI—simple disease activity index; RAID—rheumatoid arthritis impact of disease; RAQoL—rheumatoid arthritis quality of life; HAQ—health assessment questionnaire; ANA—antinuclear antibody, aCL-anticardiolipin; RF—rheumatoid factor; NSAID—Non-steroidal anti-inflammatory drugs; AH—arterial hypertension; DM—diabetes mellitus; CVI—cerebrovascular insult; TIA—transitory ischemic attack; MI—myocardial infarction; AP—angina pectoris; ESR—erythrocyte sedimentation rate.

**Table 3:** Markers and EBV infection status in RA patients and controls.

EBV Infection Parameter	Group		*p* *	Subgroups		*p* *
	RA n = 133	Control n = 50		RA A n = 80	RA B n = 53	
EBV DNA (EBNA1 and/or LMP1), n (%)	9 (6.8)	0 (0.0)	0.059 §	7 (8.8)	2 (3.8)	0.263 §
Anti-EBV-EBNA1 IgG, med (min–max)	149.0 (0.0–200.0)	195.0 (0.0–200.0)	**0.001**	**172.5 (0–200)**	**124.0 (0–200)**	**<0.001**
Anti-EBV-EBNA1 IgG	127 (95.5)	49 (98.0)	0.430 §	77 (96.3)	50 (94.3)	0.603 §
Anti-EBV-CA IgG, med (min–max)	184.0 (0.0–200.0)	199.0 (0.0–200.0)	0.104 €	194.5 (0–200)	160.0 (18–200)	**<0.001**
Anti-EBV-CA IgG	132 (99.2)	49 (98.0)	0.473	79 (98.8)	53 (100.0)	1.000 €
Anti-EBV-CA IgM, med (min–max)	0.0 (0.0–5.7)	0.0 (0.0–1.4)	**0.003**	0.0 (0.0–4.4)	0.0 (0.0–5.6)	0.132
Anti-EBV-CA IgM	26 (19.5)	1 (2.0)	**0.003** §	19 (23.8)	7 (13.2)	0.133 §
Anti-EBV-EA(D) IgG, med (min–max)	0.0 (0.0–200.0)	0.0 (0.0–90.0)	**0.023**	0.0 (0–200.0)	0.0 (0–175.0)	0.612
Anti-EBV-EA(D) IgG	26 (19.5)	3 (6.0)	**0.025** §	17 (21.3)	9 (17.0)	0.543 §
Anti-EBV-EA(D) IgM, med (min–max)	0.0 (0.0–7.3)	0.0 (0.0–2.9)	0.335	0.0 (0.0–7.0)	0.0 (0.0–7.3)	0.099
Anti-EBV-EA(D) IgM	21 (15.8)	5 (10.0)	0.317 §	16 (20.0)	5 (9.4)	0.102 §
**EBV infection status, n (%)**						
Active/recent	56 (42.1)	8 (16.0)	**<0.001** §	41 (51.25)	15 (28.3)	**0.009 §**
past	77 (57.9)	42 (84.0)		39 (48.75)	38 (71.7)	

* for the level of significance of 0.05, according to Chi-square test § or Fisher’s exact test €. Abbreviations: EBV—Epstein–Barr virus; EBNA1—Epstein–Barr nuclear antigen; LMP1—latent membrane protein; CA—capsid antigen; EA—early antigen; min—minimum; max—maximum.

**Table 4:** Disease characteristics between EBV viremic vs. non-viremic RA patients.

Disease Characteristics	RA Patients with Positive EBV DNA in Blood (Viremic RA Patients)	RA Patients with Negative EBV DNA in Blood (Non-viremic RA Patients)	*p* *
**DAS28CRP mean ± sd**	4.98 ± 1.47	5.26 ± 1.09	0.313
**CDAI med (min–max)**	14.0 (6.0–29.0)	18.0 (1.0–40.0)	0.536
**SDAI med (min–max)**	15.0 (6.0–23.0)	16.0 (0.0–63.0)	0.387
**RAID med (min–max)**	6.0 (2.0–10.0)	5.0 (1.0–18.0)	0.615
**HAQ med (min–max)**	1.1 (0.3–1.1)	1.12 (0.1–2.6)	0.250
**CRP med (min–max)**	10.1 (1.1–66.6)	15.1 (0.0–152.4)	0.402
**ANA med (min–max)**	0.00 (0.0–80.0)	40.0 (0.0–640.0)	0.565
**ACPA med (min–max)**	500.0 (3.8–500.0)	309.0 (0.0–500.0)	0.182
**RF med (min–max)**	219.0 (113.0–500.0)	149.0 (0.0–641.6)	**0.033**
**Anticardiolipin IgG**	0.0 (0.0–0.1)	0.0 (0.0–15.1)	0.148
**Anticardiolipin IgM**	0.0 (0.0–0.2)	0.0 (0.0–31.2)	0.226

* for the level of significance of 0.05. Abbreviations: DAS28CRP—disease activity score C reactive protein; CDAI—clinical disease activity index; SDAI—simple disease activity index; RAID—rheumatoid arthritis impact of disease; HAQ—health assessment questionnaire; CRP—C reactive protein; ANA—antinuclear antibody; ACPA—anti-citrullinated protein antibody; RF—rheumatoid factor.

**Table 5:** Nucleotide and amino acid residues in EBNA1 isolates from the plasma of RA patients.

	Position of Amino Acid/Codon Based on Ref. Sequence of EBV B95.8 (P-ala)										
EBV B95.8 (P-ala)	471 Gln/CAA	476 Pro/CCG	483 Glu/GAA	487 Ala/GCT	492 Ser/AGT	499 Asp/GAC	502 Thr/ACT	520 Leu/CTA	524 Thr/ACT	529 Pro/CCA	533 Leu/CTT
Patient RA33A (P-thr)	*	Gln/CAG	*	Thr/ACT	Cys/TGT	Asp/GAT	*	Leu/CTC	Ile/ATT	*	*
Patient RA35A (P-thr)	*	Gln/CAG	*	Thr/ACT	Cys/TGT	Asp/GAT	*	Leu/CTC	Ile/ATT	*	*
Patient RA37A (P-ala-sv-2)	*	*	*	*	*	Glu/GAA	*	*	Val/GTT	*	*
Patient RA41A (P-thr)	*	Gln/CAG	*	Thr/ACT	Cys/TGT	Asp/GAT	*	Leu/CTC	Ile/ATT	*	*
Patient RA62A (P-thr)	*	Gln/CAG	*	Thr/ACT	Cys/TGT	Asp/GAT	*	Leu/CTC	Ile/ATT	*	*

* The same amino acid/codon as in the reference sequence EBV B95.8 (P-ala). Gln—glutamine; Pro—proline; Glu—glutamic acid; Ser—serin; Asp—aspartic acid; Thr—threonine; Leu—leucine; Val—valine; Cys—cysteine; Ile—isoleucine.

**Table 6:** Factors associated with rheumatoid arthritis.

Factor Associated with	Multivariate Logistic Regression		
	OR/RR	95% CI OR	*p* *
**Having RA**			
Active/recent EBV infection	4.645	1.33–16.19	**0.016**
**The onset of RA**			
Active/recent EBV infection	5.471	1.28–23.35	**0.022**
**Poor control of RA**			
Active/recent EBV infection	0.451	0.21–0.98	**0.045**

* for the level of significance, according to enter logistic regression modeling, adjusted for age, gender, educational status, smoking habit and family history for RA.

## Data Availability

Data is contained within the article.
